# Host-mediated niche construction of bacterial communities in an aquatic microecosystem

**DOI:** 10.1093/ismejo/wraf233

**Published:** 2025-10-17

**Authors:** Aldo A Arellano, Journey L Prack, Kerri L Coon

**Affiliations:** Department of Bacteriology, University of Wisconsin-Madison, Madison, WI 53706, United States; Microbiology Doctoral Training Program, University of Wisconsin-Madison, Madison, WI 53706, United States; Department of Bacteriology, University of Wisconsin-Madison, Madison, WI 53706, United States; Department of Bacteriology, University of Wisconsin-Madison, Madison, WI 53706, United States

**Keywords:** niche construction, host-microbe symbiosis, mosquito, pitcher plant, microbial ecology, transient microbiota

## Abstract

Microbes coordinate homeostasis in host-associated and environmental ecosystems alike, but the connectivity of these biomes is seldom considered. Hosts exert controls on the composition and function of their internally associated symbionts, but an underappreciated modality of microbiome curation is external to the host through changes to the environmental species pool from which they recruit microbial symbionts. Niche construction theory describes how organisms alter their environment and the selective landscape of their offspring and conspecifics. We hypothesize that host-driven manipulation of environmental microbial communities is an underexplored form of this concept. Using the pitcher plant mosquito (*Wyeomyia smithii*) as a model, we tested how hosts shape microbial communities across developmental stages and gradients of pre-existing community complexity. We report three lines of evidence supporting host-mediated niche construction, leveraging amplicon sequencing and microbiota manipulation experiments with germ-free (axenic) and selectively recolonized (gnotobiotic) mosquitoes. First, single female egg-laying assays showed repeatable adult inoculation of sterile water with beneficial bacteria capable of sustaining robust larval development. Second, increasing larval density in assays inoculated with complex, field-derived microbial communities selected for environmental and host-associated bacteria that correlated with increased larval fitness. Finally, exposing axenic larvae to mixtures of parentally and environmentally derived microbiota demonstrated that prior conditioning by conspecifics enhanced offspring fitness. Although the bacterial taxa associated with mosquito structuring varied, members of the *Actinobacteriota* and *Acetobacteraceae* were consistently associated with increased fitness. Overall, our results provide an example of host-mediated niche construction to favor environmental microbial communities that positively impact host fitness.

## Graphical Abstract

**Graphical Abstract:**
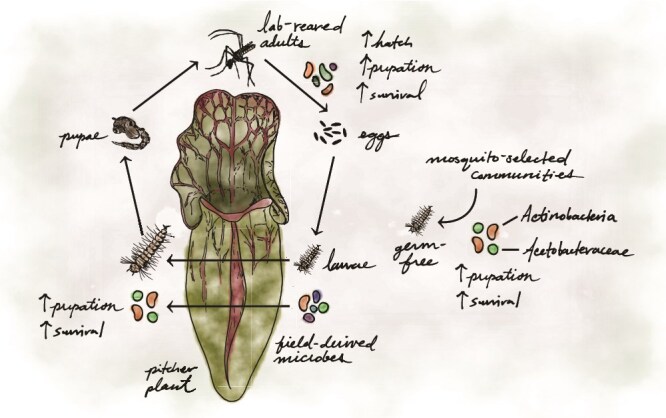

## Introduction

The external dimension of microbial curation is an understudied aspect of host–microbe interactions. Top-down control of microbial communities by “higher” trophic level eukaryotes (e.g. metazoans) is observed across diverse environments, impacting ecosystem-wide features ranging from decomposition rates to pathogen defense [1, 2]. Analogously, hosts exert influence on their internal microbial symbiont communities to maintain essential homeostatic function [3, 4]. We propose that, at the intersection of these ideas, recurrent top-down influence by hosts on environmental pools of microbiota from which host-associated taxa are recruited represents a widespread form of niche construction: the process by which organisms alter their environment and consequently, the selective pressures faced by their offspring and conspecifics [5]. Although niche construction has been explored in both macroscopic and microscopic systems (reviewed in [6]), processes that span these scales are seldom considered.

Microbial symbionts provide nutrition, facilitate adaptation, and influence physiology and behavior in insects [7–9], with relationships ranging from obligate to transient and horizontal, vertical, or mixed modes of acquisition [10–12]. As opposed to the strict vertical transmission of *Buchnera* in aphids [13] or repeatable horizontal acquisition of *Burkholderia* (*sensu lato*) by broad-headed bugs from their environment [11, 12], analogous processes structuring compositional or functional microbiome consistency in mosquitoes have not been demonstrated outside of vertical transmission of *Wolbachia* in natural and introduced settings [14]. Literature has yet to explore how host-environment interactions sustain symbiotic relationships between mosquito hosts and environmental microbial communities. Using the pitcher plant mosquito (*Wyeomyia smithii*) and its symbiosis with the purple pitcher plant (*Sarracenia purpurea*) and environmental microbiota, we tested how hosts shape microbial communities across development and gradients of ambient microbial community complexity. *W. smithii* completes its entire life cycle associated with the purple pitcher plant’s water-filled leaves (“pitchers”). Adult female *W. smithii* lay eggs in pitcher water, where larvae hatch and progress through four aquatic juvenile stages (instars) and an aquatic pupal stage before emerging as terrestrial adults. *S. purpurea* does not produce many enzymes essential for prey digestion, instead relying on a diverse community of eukaryotes and bacteria for mechanical and enzymatic breakdown of captured prey, respectively [15, 16]. Microbes are essential for *W. smithii* development, with individuals failing to progress past the first larval instar in the absence of bacteria [17]. This effect is likely mediated by similar mechanisms as elucidated by work in *Aedes aegypti*, where diverse gut microbiota provision light-sensitive B vitamins such as riboflavin that stabilize hypoxia-inducible transcription factors (HIFs) and facilitate development [18–20]. Moreover, *W. smithii* influence microbial communities through trophic cascades (*sensu* [21]) [22–25] and direct bacterivory [26, 27], ultimately contributing to shifts in microbial composition and function during primary succession in pitchers [28].

We assessed the ability of *W. smithii* to curate environmental and host-associated microbial community composition across its life cycle using three complementary approaches and leveraging 16S rRNA gene amplicon sequencing, sterile insect husbandry, and selective recolonization of germ-free (axenic) animals (gnotobiotics). We first examined how bacteria deposited by single egg-laying females in sterile environments repeatedly and positively influenced offspring development. Next, we evaluated the effects of larval density on the trajectory of host- and environmentally associated bacteria in assays inoculated with a complex field-derived microbial community. Finally, we selectively reintroduced axenic larvae to microbial communities with varying degrees of prior exposure to mosquito conspecifics and described how this variation affected larval growth. We hypothesized that top-down trophic controls by *W. smithii* on environmentally acquired microbial pools would alter offspring and conspecific fitness. Adult-associated bacteria were transmitted downstream to offspring and sustained robust larval development, increasing larval density enriched for beneficial bacterial taxa in the span of a single generation, and mosquito-selected microbial communities positively impacted larval growth. In each of our approaches, the taxa associated with mosquito selection and positive developmental outcomes included members of the *Actinobacteriota* and *Acetobacteraceae*. Our results present an example of niche construction via adaptive host curation of environmental bacterial communities across multiple life stages in a model aquatic microecosystem.

## Results

### Parentally derived taxa persist in larval water and support robust development

Our recent results support a dominant role for the pitcher environment in shaping microbiota diversity in *W. smithii* larvae, and also indicate that pitcher-associated microbiota persist in adult *W. smithii* mosquitoes [17]. Here, we evaluated whether microbiota are parentally transmitted and to what extent these bacterial communities shape offspring fitness. We accomplished this by tracking the growth and development of *W. smithii* larvae exposed only to the microbiota associated with a single egg-laying adult female and her mates (“iso-female assays”). Characterization of parental (separated by sex) and downstream (egg water or late larval water) bacterial communities, paired with measures of egg hatch rate and larval growth, then allowed us to identify bacterial communities reliably associated with egg-laying and their correlations with offspring development. We hypothesized that a subset of parental taxa would consistently colonize and persist in the rearing environment and be associated with positive offspring development.

**Figure 1:**
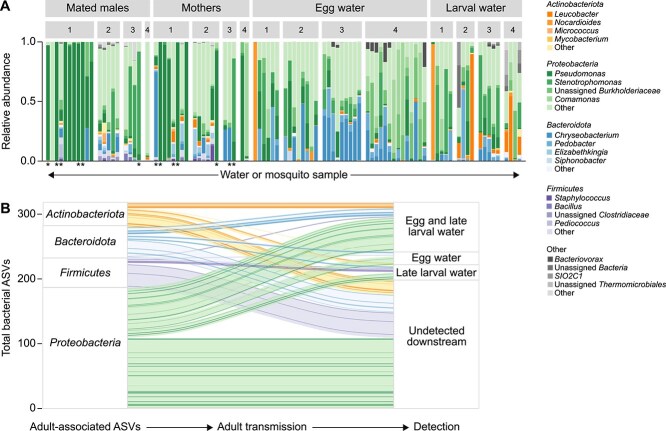
(A) Relative abundance of bacterial genera present in iso-female assays faceted by sample type (three homogenized mated males, a single homogenized female, bacterial pellet from egg water, or bacterial pellet from late larval water) and trial number (1–4). Bars of a given color represent the proportion of sequencing reads assigned to a given genus in a mosquito or water sample. The most common four genera are represented for each of the four most abundant phyla with a base color. Less common genera are collapsed to the category “Other”. Bars indicated with an asterisk correspond to adult samples that did not produce viable offspring. (B) Alluvial plot tracking retention or loss of parentally derived bacterial ASVs in downstream sample types. ASVs observed in parental samples (mated males or mothers) from the dominant four bacterial phyla are represented and colored according to phylum and commonality across the dataset. ASVs are sorted into bins corresponding to loss or observation in one or both downstream sample types (egg water and/or late larval water). Darker shades of a given color represent more common taxa across the dataset.

Sequencing of 16S rRNA gene amplicons from iso-female assays identified 524 unique ASVs across 98 samples retained for analysis following rarefaction (Supp Table 1). Of these samples, 82 were associated with iso-female lines producing a sufficient number of offspring for developmental assays and ASV tracking downstream. Of the 372 unique ASVs detected in adult samples, 130 were detected in downstream sample types (~35%) (Fig. 1A and B). Among the four major phyla represented in the sequencing dataset, we observed variable reduction in ASV diversity over transmission, with 45% *Proteobacteria*, 42% *Actinobacteriota*, 50% *Bacteroidota*, and 67% *Firmicutes* ASVs lost downstream (Fig. 1B). However, transmitted taxa comprised a dominant relative proportion of reads for all sample types (87%–99%).

**Figure 2:**
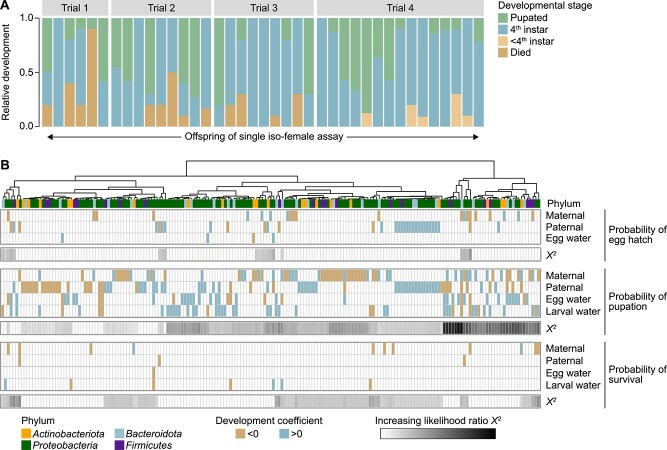
(A) Relative larval development from iso-female assays faceted by trial (1–4). Each bar represents ~10 larvae reared under sterile conditions with microbiota transmitted by a single female and her mates. Colors show the proportion of offspring that died, arrested before or at the terminal instar, or pupated. (B) Hierarchical clustering of 189 bacterial ASVs significantly associated with offspring development (hatching, pupation, survival). Clustering is based on fixed effect coefficients (positive, negative, or nonsignificant) and conditionality within a given development metric (ꭓ*^2^* coefficient). Columns represent significant ASVs from the four major phyla represented across iso-female assays, with boxes colored by effect direction (blue = positive, brown = negative) and conditionality shown as a white-to-black gradient. Correlations are grouped vertically by sample type (three males, individual female, egg water, larval water), with phylum indicated by color at the top.

We measured offspring development for a total of 39 iso-female lines restricted to parental microbial inputs. Individual maternal females displayed variable wing length (a proxy for body size) [29, 30], density of transmitted bacteria, and egg hatch rate (Supp Fig. 1A–D). Mixed-effects regressions revealed a nonsignificant positive correlation between egg water bacterial density and hatch rate (Supp Fig. 1E), a significant correlation between egg water bacterial density and larval survival (*β* = 1.35; *P* = .009, Supp Fig. 1F), and no correlation between maternal wing length and hatch rate or survival (data not shown). Additionally, we observed no significant correlation between egg water bacterial density and total number of eggs laid (data not shown). Development of larval offspring from iso-female assays suggested parentally derived taxa are frequently sufficient to support robust development. Of the 39 iso-female assays conducted, 67% resulted in no larval death, 64% yielded pupae, and 100% supported development to the final larval stage (Fig. 2A). To further explore the relationship between parentally derived microbiota and the developmental outcomes of larval offspring, we constructed mixed-effects logistic regression models correlating clr-transformed ASV counts in each sample type to the probability of attaining important life history transitions (egg hatch or pupation) and survival (Supp Table 2). We also explicitly modeled the conditionality of ASV relationships to development outcomes by sample type, using likelihood ratio tests of nested models with and without an ASV × sample type interaction term. Constraining analysis to taxa with known origin in adult samples (372 ASVs), 196 taxa were identified as significantly correlated to developmental success by at least one of our three metrics (53%). The majority of taxa with documented transfer downstream (Fig. 1B) were identified as significant (81%), with most of those taxa having only positive (47%) or context-specific correlations with offspring development (i.e. both positive and negative depending on sample type and/or development metric) (30%). A minority of adult taxa not detected downstream were also identified as significant (24%), with half of these taxa having positive (43%) or context-specific (7%) correlations with offspring development.

We conducted hierarchical clustering of all significant ASVs in the major phyla represented in our dataset (189 taxa) to identify clades of bacteria with shared correlations with mosquito offspring development (Fig. 2B). Samples clustered strongly by conditionality (Fig. 2B), with the largest group of significant taxa associated with parental mosquitoes and probability of offspring pupation, followed by parental mosquitoes and probability of egg hatch. In the former, a majority of taxa were either associated with an increased probability of pupation as ASV abundance increased (49%), or showed negative effects on pupation when increasing in some sample types, but positive effects in others (11%).

Among taxa associated with parental mosquitoes and egg hatch probability, most were associated with increased probability of hatching with increasing ASV abundance (60%; Fig. 2B). Additionally, several taxa showed negative effects on egg hatch probability in some contexts but positive effects with increasing abundance in other sample types (27%; Fig. 2B). This effect was driven largely by maternal taxa associated with decreased probability of hatching that were positively associated with probability of pupation with increasing abundance in maternal samples (86%; Fig. 2B).

Significant relationships with probability of egg hatch (58 ASVs) were uniformly positive (62%) or negative (38%) and did not vary by which sample type they were found in (Supp Table 2). Among these, relationships within the *Actinobacteriota* were predominantly positive (75%), including members of the genera *Cellulosimicrobium*, *Lawsonella*, and *Mycobacterium* (Supp Table 2). Relationships within the families *Acetobacteraceae* (*Rhodovarius* and *Roseomonas*), *Burkholderiaceae* (*Comamonas* and *Pandoraea*), *Enterobacteriaceae* (*Erwinia*, *Escherichia*, and *Pantoea*), and *Pseudomonadaceae* (*Pseudomonas*) were also predominantly positive (57%–75%; (Supp Table 2). Significant positive egg hatch relationships were observed with increasing ASV abundance in parental sample types (92%) and egg water (8%).

Significant relationships with pupation probability (188 ASVs) spanned positive (51%), negative (38%), and sample type-dependent correlations (11%). Among these, relationships within the families *Clostridiaceae* (*Clostridium*), *Acetobacteraceae* (*Asaia, Rhodovarius,* and *Roseococcus*), *Aeromonadaceae* (*Pseudaeromonas*), *Rhodocyclaceae* (*Tepidiphilus* and *Thauera*), *Sphingomonadaceae* (*Novosphingobium* and *Rhizorhabdus*), and *Xanthobacteraceae* (*Aquabacter*) were predominantly positive (50%–67%; Supp Table 2). Uniformly positive pupation relationships were observed with increasing ASV abundance in parental sample types (52%), egg water (12%), late larval water (15%), two of three sample types (19%), or—in the case of two ASVs—all sample types (a member of the genus *Cedecea* [Family: *Enterobacteriaceae*] and a member of the family *Burkholderiaceae;*  Supp Table 2).

Significant relationships with survival probability were fewer than those observed for other development metrics (20 ASVs) and were uniformly positive (25%) or negative (75%). A conserved, predominantly positive relationship (67%) was observed with increasing ASV abundance of significant taxa in the phylum *Actinobacteriota*, including members of the genera *Leucobacter* and *Corynebacterium* (Supp Table 2)*.* Uniformly positive survival relationships were observed with increasing ASV abundance in parental sample types (40%) and late larval water (60%). Late larval water taxa associated with increased probability of survival included members of the genera *Chryseobacterium* (Family: *Weeksellaceae,* mixed-effects regression; *β* = 0.48; *P* = .03) and *Bosea* (Family: *Beijerinckiaceae*, mixed-effects regression; *β* = 0.58; *P* < .001). No taxa from any iso-female species pool were associated with significant variation in offspring adult wing length or speed of offspring development (time to pupation), and neither metric differed significantly by trial (Supp Fig. 2A and B).

### Mosquito larvae reduce bacterial diversity and select for beneficial taxa in their aquatic habitat

In our next set of experiments, we assessed whether *W. smithii* larvae select for beneficial environmental and host-associated taxa when inoculated with complex bacterial communities derived from pitcher plants in the field (“community passaging assays”). In each assay, we characterized the density-dependence of top-down structuring of bacterial communities by exposing pitcher plant-derived microbes to differing densities of axenic larvae: zero larvae (“Uncolonized”), two larvae (“Field”), and 10 larvae (“High”). The “Field” colonization condition reflected the average density of larvae in unmanipulated pitchers over a field season at the Cedarburg Bog in Saukville, WI USA, as previously reported [28, 31, 32]. The composition of bacterial communities in rearing water (at 48 h and 12 days posthatch) and in terminal instar larval hosts (12 days posthatch) was then described in tandem with measures of larval survival. We hypothesized that we would observe more distinct signatures of host filtering and environmental curation with increasing larval density and that the taxa associated with these changes would be positively correlated with larval survival.

**Figure 3:**
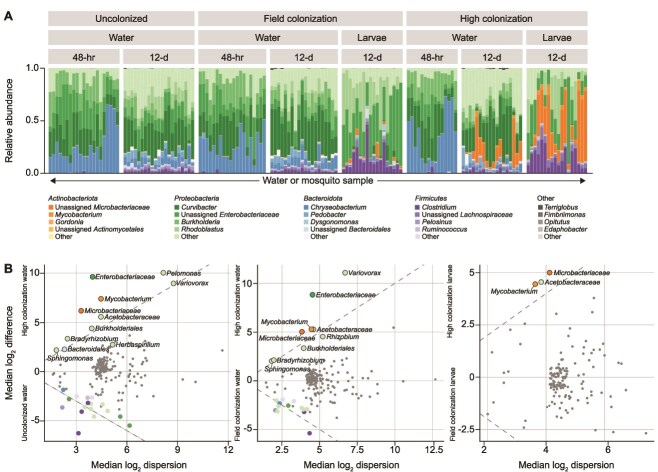
(A) Relative abundance of bacterial genera present in larval passaging assays faceted by mosquito colonization background (increasing left to right), sample type (late larval water or 2–10 homogenized larvae), and timepoint (48 h or 12 day). Bars of a given color represent the proportion of sequencing reads assigned to a given genus in a mosquito or water sample. The most common four genera are represented for each of four focal phyla with a base color. Less common genera are collapsed to the category “Other”. (B) Effect plots depicting differentially abundant bacterial ASVs enriched in larval water (*left*, *middle*) or homogenized larval samples (*right*) across mosquito colonization backgrounds at the 12 day timepoint. Each point represents a bacterial ASV with colored points indicating differential abundance. Points are colored by phylum and commonality across the dataset. Differentially abundant points enriched in “High” mosquito colonization water and larvae are further labeled by genus. Dashed lines represent equal difference (between group variation) and dispersion (within group variation). Darker shades of a given color represent more common taxa across the dataset.

Sequencing of 16S rRNA gene amplicons from rearing water and larvae identified 340 unique ASVs across 151 samples retained for subsequent analysis following rarefaction (Fig. 3A; Supp Table 3). Bacterial communities in the water of assays maintained under “High” larval density conditions had significantly lower alpha diversity at the early timepoint compared to “Uncolonized” (betta test; *β* = −0.20; *P* < .001) and “Field” (betta test; *β* = −0.25; *P* < .001) conditions (Supp Table 4), as measured by the Shannon index, which takes into account both ASV richness and evenness. At the late timepoint, all density conditions differed significantly from each other in both Shannon index values and estimates of total ASV richness, with values for both measures decreasing with higher larval density. Beta diversity measures revealed that communities diverged in composition only at the late timepoint (Supp Table 5), with water samples from assays maintained under “High” larval density conditions clustering separately from water samples derived from both “Uncolonized” assays (PERMANOVA; *t* = 27.7; *P* = .03) and those maintained under “Field”-relevant larval density conditions (PERMANOVA; *t* = 15.4; *P* = .03). Clustering was in-part driven by taxa significantly enriched in the “High” larval condition, including members of the genus *Mycobacterium* and the families *Acetobacteraceae, Microbacteriaceae,* and *Enterobactereaceae* (Fig. 3B; Supp Table 6). Increasing relative titers of several of these enriched taxa and a member of the genus *Gordonia* (mixed-effects regression; *β* = 0.17; *P* = .04) in the aquatic rearing environment were correlated with increased rate of larval survival (Supp Table 6).

Larval-associated bacterial communities exhibited significantly lower estimates of alpha diversity (Shannon index and ASV richness) relative to bacterial communities from the aquatic rearing environment at all timepoints, but did not differ significantly by larval density condition (Supp Table 4). Larval-associated communities clustered distinctly from all water samples regardless of larval condition (Supp Table 5). Larval-associated communities differed significantly between the “Field” and “High” conditions in composition (PERMANOVA; *t* = 10.7; *P* = .03). Differential abundance testing at the ASV level among larval-associated communities at “Field” versus “High” levels of colonization revealed four ASVs at significantly greater relative abundances in larvae of the “High” condition, including members of the genus *Mycobacterium* and the families *Acetobacteraceae* and *Microbacteriaceae* (Fig. 3B; Supp Table 6). Increasing host-associated relative abundances of *Mycobacterium* and *Kaistia* were correlated with increased rate of larval survival (Supp Table 6).

Bacterial community composition in water changed significantly over time for every larval density condition (Supp Table 5), with particular taxa also enriched either early or late in the experiment (Supp Table 6). However, the degree to which samples differed from the standardized starting community (beta diversity relative to the field-derived inoculum) varied significantly by sample type and larval density condition (ANOVA; *F*_7,143_: 67.3; *P* < .001; Supp Fig. 3A). Early water samples diverged similarly from the inoculating stock but were more similar to the starting community than late water samples. Late water samples containing larvae were significantly less diverged from the inoculating community than “Uncolonized” water samples. Early water samples were as similar to the starting community as larval samples regardless of larval density condition (Tukey’s HSD; *P* < .001; Supp Fig. 3A). “High” larval density water at the late timepoint exhibited significantly lower culturable bacterial density relative to “Field” and “Uncolonized” water (Tukey’s HSD; *P* < .001; Supp Fig. 3B). Total bacterial density at the late timepoint negatively correlated with probability of larval survival (mixed-effects regression; *β* = −2.42; *P* < .001).

### Reintroduction of host-selected microbial communities positively impacts mosquito development

In our final set of experiments, we sought to explore relative fitness effects of host and environmental structuring on microbial community composition by varying the relative inoculation density of parentally and environmentally derived bacterial communities into development assays containing axenic larvae and tracking larval growth. Inoculated communities were derived from one of three glycerol stocks: a “Conventional” stock containing bacteria isolated from water collected from our standard rearing colony of *W. smithii*; a “Parental” stock containing bacteria isolated from 20 iso-female assay egg sheets laid under sterile conditions; and a “Laboratory” stock containing bacteria isolated from 16 glass tubes filled with 25 ml distilled water, amended with 15 mg nonsterile cricket powder as a proxy for insect prey [28, 33], and left uncapped for 72 h in the laboratory. The “Conventional” inoculant condition corresponded to long-term mosquito and environmental effects in our lab colony; the “Laboratory” condition corresponded to acute environmental effects in the lab; and the “Parental” condition corresponded to acute adult mosquito effects. Although these communities shared a common species pool, bacterial composition was distinct across each homogenate (Supp Fig. 4) and comparisons shed light on the relationship between mosquito and environmental structuring over different timescales. We additionally utilized field-derived pitcher water homogenates with different mosquito visitation histories (“Uncolonized” or “Colonized”) to explore how parental structuring may occur in the field. Water samples were collected for characterization of the aquatic bacterial community at first pupation in each assay. We hypothesized that inoculating communities previously exposed to larval or adult selection would positively impact larval fitness, particularly when these communities contributed a larger relative density to culture inocula.

**Figure 4:**
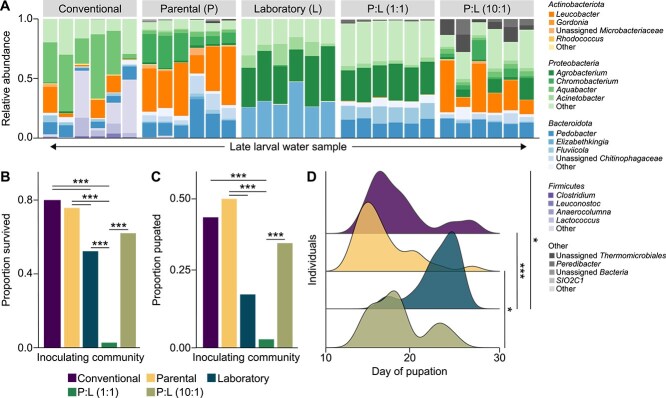
(A) Relative abundance of bacterial genera present in community inoculation assays faceted by treatment. Bars of a given color represent the proportion of sequencing reads assigned to a given genus in a late larval water sample for a given well of a six-well plate gnotobiotic development assay. The most common four genera are represented for each of four focal phyla with a base color. Less common genera are collapsed to the category “Other”. (B) Proportion of larvae in each gnotobiotic community background that survived the 27 day period in which development was recorded. (C) Proportion of larvae in each gnotobiotic community background that pupated during the 27 day period in which development was recorded. (D) Ridgeline stacked density plots for time to pupation across gnotobiotic community backgrounds. Curve height indicates a greater number of individuals pupating at a given point along the horizontal axis. Inoculation source (conventional, parental, laboratory, or mixed inocula of parental and laboratory) is indicated by color. Asterisks indicate significant differences in proportional development (Barnard’s test) or time to pupation (Dunn’s test). For overlapping asterisks the more significant annotation supersedes the other. * *P* < .05, ** *P* < .01, *** *P* < .001.

Sequencing of 16S rRNA gene amplicons from stocks and water samples from development assays inoculated with single stocks or varied relative concentrations of stocks identified 640 unique ASVs across 64 samples retained for subsequent analysis following rarefaction (Fig. 4A; Supp Fig. 5A; Supp Table 7). Assays receiving “Parental”, “Laboratory”, or “Conventional” inocula alone differed globally in their alpha diversity (Shannon index and ASV richness; Supp Table 8), with samples from “Parental”-inoculated assays exhibiting significantly greater ASV richness (betta test; *β* = 23.65; *P* = .01) and Shannon index (betta test; *β* = 1.09; *P* < .001) relative to “Laboratory”-inoculated assays. Mixed community inoculation (1:1 and 10:1 solutions of “Parental:Laboratory” stocks) also produced samples with greater richness values relative to samples from assays inoculated with the “Laboratory” stock alone (Supp Table 8). Samples from assays inoculated with partially field-derived inocula (1:1 and 10:1 solutions of “Parental:Uncolonized” stocks) exhibited higher richness than either single inoculum (Supp Table 8). Samples from “Colonized” assays also exhibited significantly higher richness than samples from “Uncolonized” assays (betta test; *β* = 31.85; *P* < .001).

Analysis of beta diversity revealed that the composition of bacterial communities in late larval water samples from “Parental”-, “Laboratory”-, and “Conventional”-inoculated assays all differed significantly from each other (Supp Table 9). The composition of samples from “Parental”-inoculated assays differed significantly from those collected under mixed inoculation conditions in a dose-dependent manner (more similar to samples with a higher relative inoculum of the “Parental” stock; Supp Table 9). Analogously, the composition of samples from “Laboratory”-inoculated assays differed significantly from those collected under mixed inoculation conditions in a dose-dependent manner (Supp Table 9). For samples from assays inoculated with partially field-derived inocula (“Parental:Uncolonized”), the 1:1 condition differed compositionally from both singular inocula (Supp Table 9), but the 10:1 condition did not. Composition of samples from these mixed conditions differed significantly from each other (PERMANOVA; *t* = 5.4; *P* = .048) as well as samples between “Uncolonized”- and “Colonized”-inoculated assays (PERMANOVA; *t* = 6.1; *P* = .048).

Relative to larvae in “Conventional”-inoculated assays, larvae in “Laboratory”-inoculated assays exhibited significantly lower rates of survival and larvae in assays inoculated with a 1:1 solution of “Parental:Laboratory” stocks exhibited significantly lower rates of both survival and pupation (Barnard’s test; *P* < .05; Fig. 4B and C). Rates of survival and pupation were also significantly higher for larvae in assays inoculated with the “Parental” stock relative to those inoculated with the “Laboratory” stock or a 1:1 solution of the “Parental” and “Laboratory” stocks, but were rescued by inoculation with a 10:1 solution (Fig. 4B and C). Larvae in “Conventional”-, “Parental”-, and “Parental:Lab (10:1)”-inoculated assays all pupated significantly earlier than larvae in “Laboratory”-inoculated assays (Fig. 4D). No field-derived conditions differed significantly from one another or from the “Parental” condition in survival or pupation (Supp Fig. 5B and C), though larvae in “Parental”-inoculated assays pupated significantly sooner than those inoculated with a 1:1 mix of “Parental” and “Uncolonized” stocks. Larvae in “Uncolonized”-inoculated assays also pupated significantly sooner than larvae in “Colonized”-inoculated assays (Supp Fig. 5D). Linear models identified a significant relationship between inoculation treatment and wing length of adult offspring, even when accounting for differences in larval survival between treatments (*t* = −2.7; *P* = .01). Adult female mosquitoes from “Conventional”- (*t* = 2.9; *P* = .006) and “Lab”-inoculated (*t* = 2.6; *P* = .013) assays were significantly larger than those generated by the “Parental” background.

**Figure 5:**
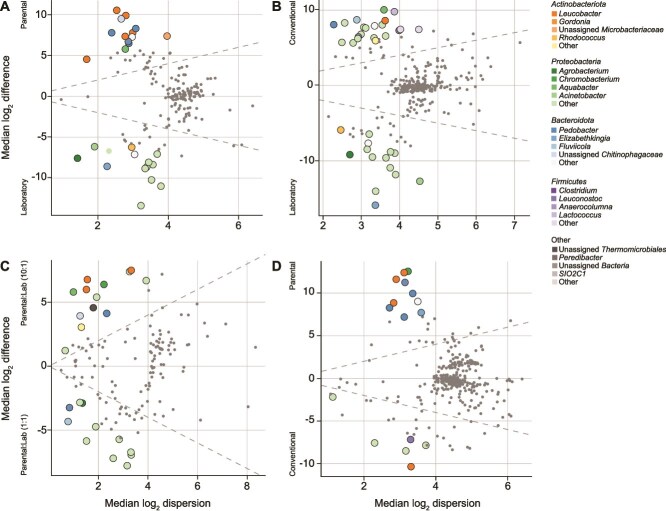
(A–D) Effect plots depicting differentially abundant bacterial ASVs enriched in pairwise comparisons of late larval water across gnotobiotic community backgrounds. Each point represents a bacterial ASV and colored points were conservatively identified as differentially abundant. Points are colored by phylum and commonality across the dataset. Dashed lines represent equal difference (between group variation) and dispersion (within group variation). Darker shades of a given color represent more common taxa across the dataset.

Comparing “Parental”- and “Laboratory”-inoculated assays, 13 and 14 ASVs were identified as enriched, respectively (Fig. 5A; Supp Table 10),. “Parental”-enriched ASVs included members of the genera *Leucobacter*, *Pedobacter*, *Aquabacter*, and *Fluviicola*, as well as the families *Microbacteriaceae* and *Chitinophagaceae*. 20 taxa were significantly enriched in “Conventional”- relative to “Laboratory”-inoculated assays, including three ASVs that were also enriched in “Parental”-inoculated assays (Fig. 5B). 19 ASVs were enriched in “Parental:Lab (1:1)”-inoculated assays and 15 in “Parental:Lab (10:1)”-inoculated assays. Taxa associated with improved larval fitness in the latter condition included six “Parental”-enriched taxa and members of the genera *Nocardioides*, *Chromobacterium*, *Pigmentiphaga*, and *Escherichia* (Fig. 5C). Although both “Conventional”- and “Parental”-inoculated assays supported similar larval development, six ASVs were enriched in the former and 10 in the latter. All “Parental”-enriched taxa were represented in other pairwise comparisons, with the exception of members of the genera *Chryseobacterium* and *Elizabethkingia* (Fig. 5D). Samples from assays with partially field-derived inocula did not exhibit any differentially abundant taxa, but “Colonized”-inoculated samples were enriched in six taxa relative to “Uncolonized”-inoculated samples (Supp Fig. 6).

## Discussion

This study leveraged three independent empirical approaches to demonstrate host-mediated niche construction of environmental bacterial communities in an aquatic microecosystem. These assays spanned developmental stages (from larvae to adults) and a range of ambient bacterial community diversity (sterile to complex). We observed taxonomically similar clades of putatively beneficial taxa persisting during association with *W. smithii* that were correlated with increased host fitness and rescued development when selectively reintroduced to axenic individuals. These results demonstrate a role for animal hosts in adaptively structuring environmental pools of potential microbial symbionts and also suggest an important role for *W. smithii* in community assembly and succession in aquatic *S. purpurea*-associated inquiline communities.

The goal of our iso-female assays was to examine how the bacterial community associated with egg-laying in *W. smithii* is reliably transmitted to offspring and affects larval development. Previous studies in the yellow fever mosquito (*A. aegypti*) suggest that maternally derived bacteria support larval mosquito development [34], but did not identify particular taxa conferring benefits in mixed communities. We found that bacteria transmitted during egg-laying improve egg hatch rate and larval survival in *W. smithii*, with several commonly transmitted taxa associated with increased larval fitness. Although several parentally transmitted taxa were uniformly associated with negative fitness outcomes, the majority of taxa correlated with negative relationships exhibited context-dependent improvement of larval fitness, conferring benefits only in specific sample types or at certain points in development. Overall, although there is high variation in adult-associated bacterial communities, a consistent set of influential and predominantly beneficial taxa successfully establish downstream. In addition, whereas previous studies have emphasized the importance of maternal taxa, increasing relative abundance of several paternal bacteria was associated with increased offspring success. These associations may arise through direct effects on adult male fitness, interactions with female microbiota during mating, or inoculation of egg-laying sites.

Several positively influential bacterial taxa in our iso-female assays are closely related to well-characterized mosquito-associated taxa or taxa consistently associated with insects. For example, *Asaia,* which was associated with increased pupation when present in mothers and transferred to *W. smithii* offspring, has also been observed within the midgut, reproductive tracts, and salivary glands in several other mosquito species and across phylogenetically distant orders of insects [35, 36]. *Asaia* are acetic acid bacteria capable of nitrogen fixation and previously implicated in immunoregulatory function in *Anopheles* mosquitoes [37, 38]. Developmental relationships with *Asaia* spp. have been previously demonstrated in larval mosquitoes [39], with beneficial roles likely mediated through interactions with the vertically transmitted mosquito microbiome [40, 41]. Other closely related *Acetobacteraceae* were also positively correlated with larval development, including*, Roseococcus* and *Roseomonas*, members of which have been previously explored in the context of symbiotic interactions within the *Microcyctis* a*eruginosa* phycosphere and antipathogen defense in the human skin microbiome, respectively [42, 43]. *Burkholderiaceae*, *Enterobacteriaceae*, and *Weeksellaceae* were also commonly and positively associated with development in iso-female larval assays. Many members of the family *Burkholderiaceae* have been found to be both mutualistic or pathogenic in insect symbioses and in *W. smithii* in particular [17, 28, 44]. *Enterobacteriaceae* and *Weeksellaceae* promote growth in other dipteran hosts and are commonly present in mosquito gut microbiomes [45, 46]. *Elizabethkingia—*a member of the *Weekselllaceae—*has been demonstrated to positively impact the development and fitness of *A. aegypti* mosquito larvae [34] and is commonly found in pitcher communities where *W. smithii* larvae develop [17, 28].

Actinobacteria were also commonly associated with positive larval development outcomes in our iso-female assays. This group of bacteria is well-known for the production of bioactive metabolites, such as antibiotics, enzymes, and signaling molecules. Further, these taxa play well-documented symbiotic antifungal or antibiotic roles across diverse insect systems [47–50]. Of those actinobacterial taxa specifically correlated with positive developmental outcomes, *Micrococcus* has previously been found to induce antimicrobial activity in *A. aegypti* (Diptera: Culicidae) [51], *Leucobacter* has been shown to be instrumental in host-defense mechanisms for *Caenorhabditis elegans* [52], and *Cellulosimicrobium* has been implicated in the degradation of recalcitrant plant matter in insect diets [53]. The higher relative abundance of actinobacteria in egg water and late larval water samples relative to adult samples suggests that these taxa are transferred with high fidelity at low density, but nonetheless play an important role later in larval development. The context-dependence of bacterial community composition aligns with our measures of conditionality, as many positive associations between increasing taxon abundance and offspring fitness were specific to particular sample types or developmental metrics. This suggests that certain mosquito-associated bacterial community dynamics over developmental time promote mosquito growth.

The goal of our larval passaging assays was to examine how larval density influences changes in field-derived diverse microbial communities. The presence of *W. smithii* larvae changed the community in the rearing environment in a density-dependent manner, revealing that over time certain bacterial taxa are favored and become more relatively abundant. This directionality and density-dependent effect of larvae is consistent with our previous reports of *Wyeomyia*-bacterial diversity relationships in the field [28]. Two days after community inoculation, and persisting through the experiment, water samples from higher larval densities exhibited lower ASV evenness, supporting early larval-mediated selection. This effect was observable in water samples with lower larval densities later in the experiment, suggesting larval-mediated effects were offset in time, but still occurred. Larvae experiencing higher densities of conspecifics were more distinct from their surrounding environment and more similar to each other than those with lower conspecific density. Increased external selection on environmental bacterial communities was thus associated with increased selectivity for host-associated taxa. Density-dependence and time offset of change in the environmental bacterial community broadly suggest mechanisms underlying larval selection, such as variation in dissolved nutrient concentrations (e.g. nitrogen supplementation through waste production, as previously described [28]), pH, or differential survival through larval gut transit. Tandem measures of water chemistry through larval selection and profiling of bacterial viability through the larval gut could elucidate potential mechanisms of larval-mediated selection.

Among taxa positively associated with favorable developmental outcomes in larval passaging experiments, members of the genus *Gordonia* are found in the guts of sand flies (*Phlebotomus argentipes*) and on the eggs of black soldier flies (*Hermetia illucens*), where they are associated with increased female egg-laying [54, 55]. In our assays, this taxon was associated with higher probability of larval survival together with other members of the *Actinobacteriota*, highlighting a conserved taxonomic signal of mosquito enrichment of taxa associated with heightened fitness. Similarly, members of the *Acetobacteraceae* were associated with larval enrichment and increased larval survival. Given that iso-female assays and larval passaging experiments had highly distinct species pools (laboratory colony and field collections, respectively), distinct focal life stages (adults and larvae), and complexity of pre-existing communities (sterile and complex), our observation of conserved taxonomic signals of adaptive community curation is notable.

Although our iso-female laboratory assays explored individual-level variation in bacterial transmission by adult mosquitoes during visitation, they likely did not fully represent levels of adult visitation in the field [56–58]. Thus, the goals of our third approach were to (i) consider the composition and quality of bacterial communities inoculated by repeated maternal visitations, and (ii) determine the relative contributions of differing bacterial species pools (mosquito-associated vs. the ambient environment) to larval development. We observed robust development of gnotobiotic larvae inoculated with communities transmitted by adults (“Parental”) or exposed to long-term mosquito selection (“Conventional”). Laboratory-derived communities that had not experienced mosquito selection of any sort were comparatively worse at sustaining larval development. When combining the parentally and laboratory-derived inoculants at a 1:1 ratio, larval outcomes were greatly negatively affected, with resultant communities largely resembling laboratory-derived microbes. This suggests a failure of parentally derived microbes to establish when in competition with an existing community of equal density. In contrast, a tenfold increase in parentally derived microbiota relative to laboratory-derived microbiota rescued larval development to levels observed under conspecific structuring, with resulting communities again dominated by parental bacteria. Taken together with the observation that *W. smithii* females preferentially lay eggs into newly opened pitchers [56–58] that harbor a low-diversity, early succession resident microbial community in the field [25], there may be an adaptive benefit to arriving at pitchers early to bias community composition trajectories to favor taxa which support larval development. Our observation of long-lasting parental structuring is consistent with previous research in the *S. purpurea* system implicating dispersal, historical contingency, and priority effects in temporal compositional trends [33, 59, 60]. Consistent with larval passaging and iso-female assays, assays inoculated with parentally derived microbes alone or at a relatively higher density were enriched in members of the *Actinobacteriota*. The actinobacterial association with positive larval development across all experiments is supportive of a nonspecific beneficial relationship between actinobacteria and *W. smithii* maintained by top-down host processes by both adults and larvae.

Gnotobiotic development assays inoculated with mixtures of field- and parentally-derived bacteria did not exhibit the same dose-dependent modulation of late larval water bacterial community composition and host fitness. This is in-part related to the fact that field inocula alone supported robust larval development through adulthood and that parental additions did not contribute to community composition as much as they did to laboratory-derived communities. Additionally, we did not observe negative synergy when mixing parental and field inocula as occurred in parental co-inoculation with the ambient laboratory community. Given that both parentally and laboratory-derived communities have in-part been shaped by the laboratory setting, it is possible that there is a greater degree of niche overlap in the microbes present in these inoculants. Microbes with higher niche overlap may compete more readily under acute changes to community composition and/or environmental stress [61] or engage in inter-microbial allelopathic interactions to the detriment of their host [62, 63]. In contrast, discrete resource partitioning between parentally and field-derived microbes may underlie more stable bacterial coexistence [64]. Although fitness differences were not observed in gnotobiotic assays inoculated with colonized and uncolonized pitcher fluid homogenates, similar hallmark *Actinobacteriota* were enriched in late larval water communities conditioned by previous *W. smithii* visitation. Moreover, bacterial richness was greater in late larval communities inoculated with colonized pitcher homogenate and culturable bacterial densities were greater in pitcher fluid relative to the uncolonized field condition. This demonstrates parentally mediated shifts to bacterial communities in a field background for pitchers early in primary succession and suggests that though long-term structuring may lead to lower diversity of mature communities [28], parental seeding of newly opened pitchers may acutely increase bacterial titers and diversity.

Mosquito-microbiota interactions and the role they play in mosquito fitness remain understudied in mosquito species with diverse ecologies and life history strategies. Integrating these species into the study of vector biology and insect-microbe interactions writ-large offers insight into evolution and function of animal-microbe interactions in natural ecosystems. Niche construction presents a framework in which to study these and other complex host–microbe interactions that involve host influences on their environment that in-turn affect their fitness and function. Examples of niche construction are commonly constrained to cases of active physical manipulation, but lasting chemical and biotic legacies imparted by an organism’s trophic and metabolic activities on the environment are at play in diverse ecosystems and constitute an important eco-evolutionary process that will often satisfy historically outlined conditions of niche construction: (i) significant environmental change, (ii) a concomitant modification of selective pressures, and (iii) a detectable evolutionary response [65]. In the case of *W. smithii*, although biotic modification may be incidental to basic features of behavior (e.g. adult egg-laying, larval feeding, and/or larval waste production), these processes are themselves selective sieves that repeatedly subset bacterial communities and are correlated with increased offspring fitness, rescuing growth upon selective reintroduction to germ-free recipients in a dose-dependent manner. While functional characterization remains to elucidate specific mechanisms by which mosquito-mediated niche construction may occur in natural settings, using a laboratory model of *W. smithii* and contrasting low diversity laboratory and high diversity field-derived microbial communities, we highlight the ability of top-down processes carried-out by mosquitoes across development to influence community composition in a model aquatic microecosystem*.* The feedback between hosts and their ambient microbial environment is an understudied modality by which hosts may externally shape their microbiome. The results from our series of experiments reiterate the importance of studying symbioses in their ecological context and of considering transient host–microbe interactions to elucidate cryptic relationships between hosts and diverse consortia of microbes.

## Materials and methods

### Iso-female assays

To characterize the variability of egg-laying associated bacterial communities and their ability to sustain larval development, we conducted assays under sterile conditions in which a single female and three males were the sole source of microbial input for cohorts of 10 larvae. Assays spanned four independent trials, ranging from 12 to 24 assays per trial due to variable mating success and egg-laying in a given trial. Conventionally reared pupae (**Supp Methods**) were sexed using genital lobe morphology under a dissecting microscope (Leica S9E) and placed in 8 ml of sterile water within sterile enclosed cages. Following adult emergence, the pupal water was removed and a half-sheet of autoclaved Whatman filter paper (Cytiva) provisioned for egg-laying. Adults also had continuous access to 0.2 μm filtered 5% sucrose.

To accommodate different adult emergence times for male and female *W. smithii*, duration of female sexual maturation [66, 67], and duration of egg-laying [68], egg sheets were sterilely harvested 48 h after female emergence [66]. Due to variation in female emergence, the total duration of a given iso-female assay varied between 4 and 5 days to standardize adult mating time. Adults were collected and stored at −20°C for subsequent wing measurement and DNA extraction performed as previously optimized for kit-based isolation of mosquito-associated bacterial DNA ([69]; **Supp Methods**). Eggs were resuspended in 20 ml sterile water with 15 mg of autoclaved cricket powder (JR Unique Foods Ltd) [33]. After 72 h, egg counts and viability were recorded, and water samples collected for enumeration of colony forming units (CFUs) on Reasoner’s 2A medium (R2A; Difco BD, Sparks, MD) and DNA extraction (5 ml). Ten larvae were randomly transferred to glass tubes (Kimax) containing 15 ml of sterile water and 10 ml residual egg water. This total volume falls within the range of pitcher volumes observed in the field [28], with the 10 ml egg water inoculum representing all egg-laying fluid remaining after DNA sample collection. 10 larvae were used for consistency with previous gnotobiotic experiments and in-field manipulations undertaken in this system [17, 28]. Axenic controls (in which eggs were surface sterilized and larvae maintained on a sterile diet) were established in parallel to ensure no bacterial contamination occurred. Axenicity was confirmed by lack of larval development, plating of any culturable microbes, and PCR [17]. Development was recorded daily for 27 days, after which the rate of pupation rapidly decreases under laboratory rearing conditions [17]. At first pupation, 5 ml of water were collected for DNA extraction. Pupae were removed as recorded and maintained in sterile water prior to adult emergence. All iso-female experiments were maintained under standard colony conditions for temperature, photoperiod, and relative humidity (**Supp Methods**). Wings from parental adults and offspring were slide-mounted and measured as a proxy for adult size using a Leica S9i digital stereo microscope (standard length measured was from the axillary incision to the tip, excluding fringe, on the right forewing) [29, 30]. Subsequent image analysis was conducted using LAS EZ image capture software and ImageJ [70].

### Community passaging under varying larval density

Field-derived microbial communities from mature pitcher fluid were passaged through varying densities of *W. smithii* larvae to assess larval density effects on bacterial community assembly. Eggs were first collected from the standard rearing colony and surface sterilized, as previously described [17]. After 72 h, hatched axenic larvae were transferred to glass tubes (Kimax) containing 25 ml sterile distilled water. Larvae were added according to one of three larval density conditions: “Uncolonized” (zero), “Field” (two), and “High” (10). The “Field” condition reflected average larval density for pitchers at Cedarburg Bog in Saukville, WI USA, as previously reported [28, 31, 32]. Each tube also received 10 μL of glycerol stock derived from late successional pitcher fluid in unmanipulated pitchers from Cedarburg Bog (~10 000 CFU/ml) ([28]; **Supp Methods**) and ~30 mg of autoclaved cricket powder. Axenic controls were established in parallel and axenicity was confirmed by lack of larval development, plating of any culturable microbes, and PCR [17]. All larval passaging experiments were maintained under controlled colony conditions for temperature, photoperiod, and relative humidity (**Supp Methods**). At 48 h and 12 days postinoculation, 5 ml of fluid were collected from each tube for DNA extraction. At the late timepoint, all remaining larvae were collected and their developmental stage noted prior to storage at −20°C for subsequent DNA extraction. Culturable bacterial density in rearing water at 12 days was determined in each tube by plating on R2A medium.

### Mixing of parentally and environmentally derived microbial communities

To determine the relative contributions of microbial species pools to mosquito development outcomes, stocks of several relevant communities were generated, including: “Conventional”, derived from 25 ml of standard colony rearing water; “Parental”, the concentrated rinsate of 20 iso-female assay egg sheets; and “Laboratory”, concentrated fluid from 16 glass tubes (Kimax) filled with 25 ml distilled water, amended with 15 mg nonsterile cricket powder, and left uncapped for 72 h in the laboratory. Field homogenates from pitchers with (“Colonized”) and without (“Uncolonized”) prior mosquito visitation were also generated (**Supp Methods).** In all cases, bacteria were isolated from homogenates by stepwise centrifugation, first for 20 min at 3000 rcf (× *g*) to accommodate large volumes, and next for 20 min at 20 000 rcf (× *g*). The concentrated material was mixed 1:1 with 0.2 μm filter-sterilized 40% glycerol and stored at −80°C. For each assay, eggs from the standard rearing colony were surface-sterilized [17] and a given assay was conducted in six replicate wells of a cell-culture plate, each containing 10 axenic larvae, 5 ml sterile distilled water, and ~10 mg autoclaved cricket powder. Glycerol stock amendments were normalized to a total density of ~900 000 CFU/ml (average culturable bacterial density of uncolonized young pitchers in the field Supp Fig. 7). Assays assigned to the “Laboratory,” “Parental,” and “Conventional,” treatment levels (laboratory-derived) or “Colonized” and “Uncolonized” treatment levels (field-derived) were inoculated with a single stock. Mixed community inoculations were carried out by maintaining an inoculation density of ~900 000 CFU/ml, but titrating the relative contributions of single inocula (e.g. ~450 000 CFU/ml from each of two stocks for a 1:1 ratio). Axenic controls were established in parallel and axenicity was confirmed by lack of larval development, plating of any culturable microbes, and PCR [17]. For the proceeding 27 days, wells were tracked for larval development. A 1 ml water sample was collected at the point of first pupation in a given well for subsequent DNA extraction. Pupae and adults were treated as detailed above for collection and wing measurement. All gnotobiotic experiments were maintained under controlled colony conditions for temperature, photoperiod, and relative humidity (**Supp Methods**).

### Sequencing data analysis

Library preparation and sequence processing were carried-out using dual-indexed one-step amplification of the V4 region of the bacterial 16S rRNA gene with barcoded universal bacterial primers 515F (5′-GTGCCAGCMGCCGCGGTAA-3′) and 806R (5′-GGACTACHVGGGTWTCTAAT-3′) as previously described (**Supp Methods**) [71]. Alpha diversity metrics were estimated using the R packages “breakaway” and “DivNet” [72, 73] and hypothesis testing for alpha diversity metrics was conducted using the *betta()* function in the “breakaway” package [74]. Beta diversity ordinations were performed on an Aitchison distance (dissimilarity) matrix using principal component analysis (PCA) after centered log-ratio (clr) transformation of the feature abundance table using the R package “microbiome” [75]. Permutational multivariate analysis of variance (PERMANOVA) was carried-out using the *adonis()* function in the R package “vegan” [76] and pairwise tests were corrected using the Bonferroni method [77]. Differences in variance were assessed using the *betadisper()* function in “vegan” [76]. Differentially abundant taxa across different categorical or continuous covariate groups were identified using the R package “ALDEx2” [78] and all generated *P* values were subjected to Benjamini-Hochberg false discovery rate (FDR) adjustment.

### Other statistical analyses

Mixed-effects regressions were used for correlating continuous predictors with continuous or proportional response variables while accounting for random effects of independent experimental trials, using the “lme4” package in R. Regression analysis of sequencing data used the clr-transformed feature table as input and resulting *P* values were subjected to Benjamini-Hochberg FDR adjustment. Hierarchical clustering of model coefficients was performed on the basis of coefficient sign for models using different underlying data normalization (i.e. clr-tranformation on data subsets) and sign and magnitude for models normalized equivalently (i.e. global clr-transformation). Model conditionality was assessed using likelihood ratio tests of nested models with and without interaction terms; the computed ꭓ*^2^* statistic represents statistical support for interaction term significance. For distributions meeting the assumptions of parametric statistics, differences in continuous response variables for two discrete predictors were determined using Welch’s *t*-tests. For three or more discrete predictors, differences were determined using an omnibus ANOVA followed by a pairwise Tukey–Kramer HSD test with Bonferroni adjustment. For distributions failing to meet the assumptions of parametric statistics, differences in continuous response variables for three or more discrete predictors were determined using an omnibus Kruskal–Wallis test followed by pairwise Dunn’s tests with Benjamini-Hochberg FDR adjustment. Proportional survival and pupation data were compared across treatment groups using an omnibus Chi-squared test followed by Bonferroni-adjusted pairwise Barnard’s tests for comparisons of interest.

## Supplementary Material

supp_fig_1_wraf233

supp_fig_2_wraf233

supp_fig_3_wraf233

supp_fig_4_wraf233

supp_fig_5_wraf233

supp_fig_6_wraf233

supp_fig_7_wraf233

Supplemental_Table_1_wraf233

Supplemental_Table_2_wraf233

Supplemental_Table_3_wraf233

Supplemental_Table_4_wraf233

Supplemental_Table_5_wraf233

Supplemental_Table_6_wraf233

Supplemental_Table_7_wraf233

Supplemental_Table_8_wraf233

Supplemental_Table_9_wraf233

Supplemental_Table_10_wraf233

Supplemental_Material_revision_3_wraf233

## Data Availability

Raw Illumina reads are available in the NCBI Sequence Read Archive (https://www.ncbi.nlm.nih.gov/sra) under BioProject ID PRJNA1216663. Scripts and intermediate files used for analysis and figure generation are available in the Coon laboratory’s GitHub repository (https://github.com/kcoonlab/wyeomyia-niche-construction). All other data generated by this study are available as Supporting Information herein.
