# Supplementary figures and images for: Host-mediated niche construction of bacterial communities in an aquatic microecosystem
Source: ISME J. 2025 Oct 17;19(1):wraf233. doi: 10.1093/ismejo/wraf233 (PMC12596266; doi:10.1093/ismejo/wraf233)

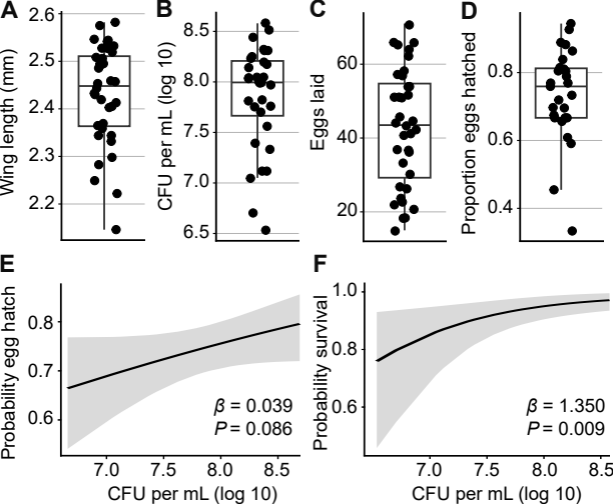

Supplement: supp_fig_1_wraf233 [file supp_fig_1_wraf233.pdf]

**A**

Time to pupation (days)

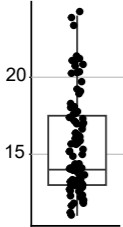**B**

Female offspring

wing length (mm)

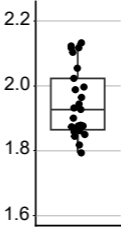

Male offspring

wing length (mm)

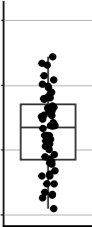

Supplement: supp_fig_2_wraf233 [file supp_fig_2_wraf233.pdf]

**A** $\beta$ -Diversity relative to starting community (a.u.)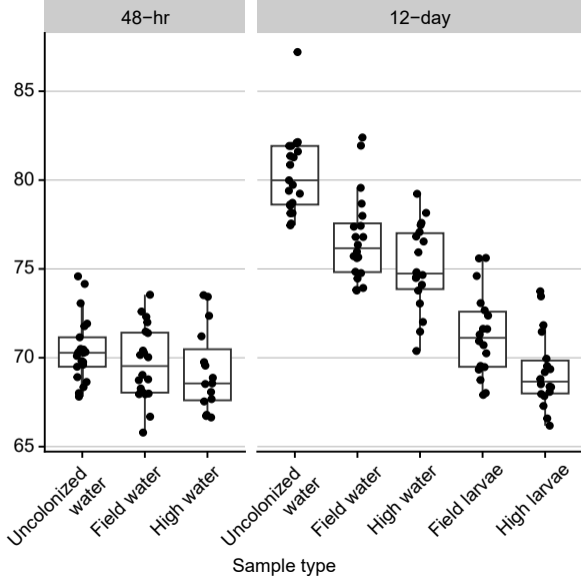**B**

log(CFU/mL)

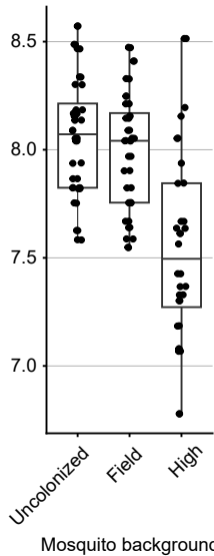

Supplement: supp_fig_3_wraf233 [file supp_fig_3_wraf233.pdf]

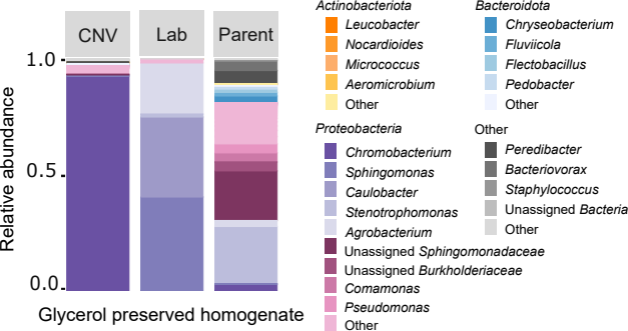

Supplement: supp_fig_4_wraf233 [file supp_fig_4_wraf233.pdf]

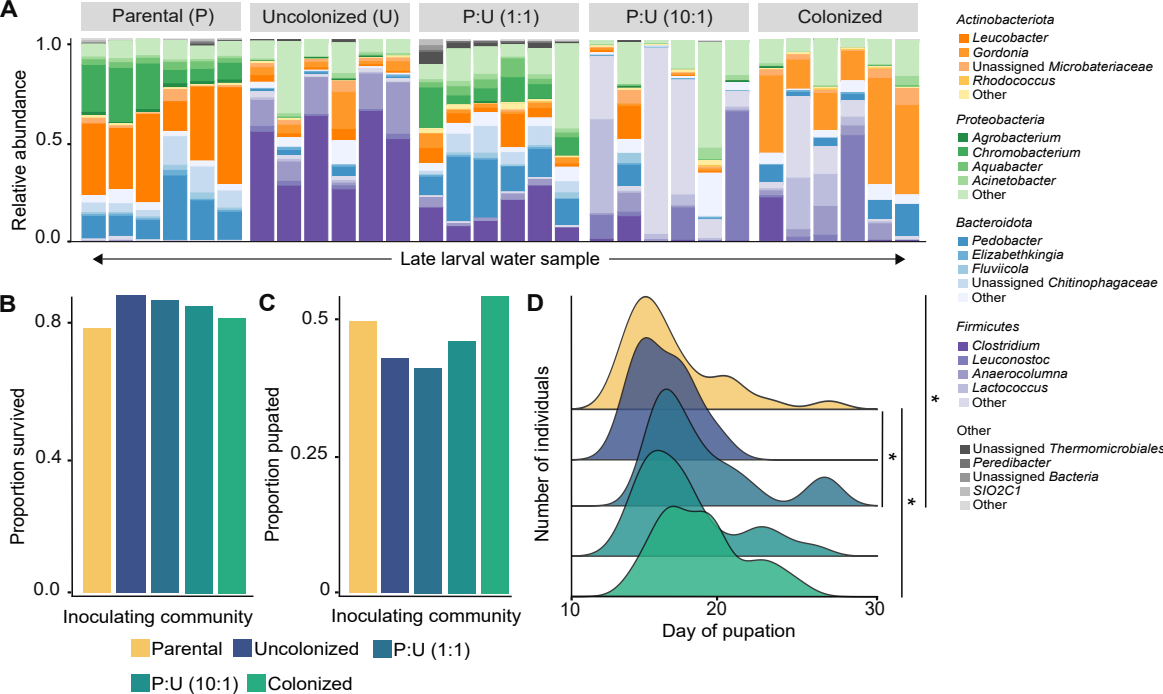

Supplement: supp_fig_5_wraf233 [file supp_fig_5_wraf233.pdf]

Median Log<sub>2</sub> Difference

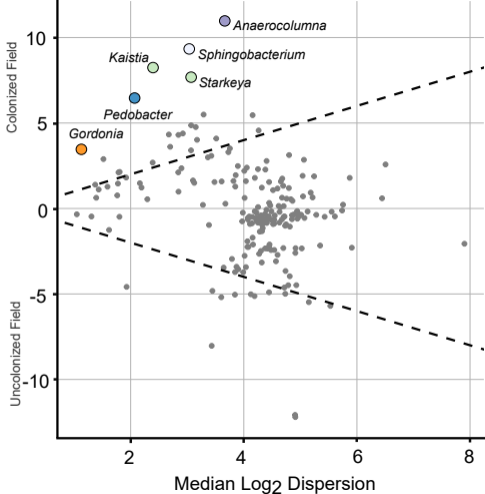

Supplement: supp_fig_6_wraf233 [file supp_fig_6_wraf233.pdf]

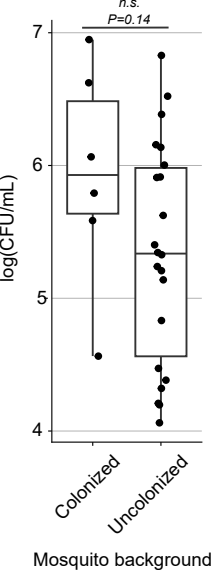

Supplement: supp_fig_7_wraf233 [file supp_fig_7_wraf233.pdf]
