# Supplementary material for: Host-mediated niche construction of bacterial communities in an aquatic microecosystem
Source: ISME J. 2025 Oct 17;19(1):wraf233. doi: 10.1093/ismejo/wraf233 (PMC12596266; doi:10.1093/ismejo/wraf233)
Supplement: Supplemental_Material_revision_3_wraf233 [file supplemental_material_revision_3_wraf233.docx]

***Supplemental Material for:***

***Host-mediated niche construction of bacterial communities in an aquatic microecosystem***

***Aldo A. Arellano^1,2*#^, Journey L. Prack^1*^, Kerri L. Coon^1#^***

***^1^Department of Bacteriology, University of Wisconsin-Madison, Madison, WI USA***

***^2^Microbiology Doctoral Training Program, University of Wisconsin-Madison, Madison, WI USA***

***^*^Equal contribution***

***^#^Correspondence: Aldo A. Arellano,*** [***aldo.a.arellano@gmail.com***](mailto:aldo.a.arellano@gmail.com)***,
Kerri L. Coon,*** [***kerri.coon@wisc.edu***](mailto:kerri.coon@wisc.edu)

**Supplemental methods**

*Mosquito husbandry*

The laboratory population of *W. smithii* used to generate eggs and for iso-female experiments was maintained in a temperature-controlled incubator (Percival) at 25 °C, > 70% relative humidity, and 16-h light: 8-h dark photoperiod. All mosquito experiments (iso-female development, larval passaging, and gnotobiotic assays) were also conducted under these controlled temperature, photoperiod, and relative humidity conditions. As previously described, larvae hatched from eggs laid ~72 hours previously were used to start each larval cohort (four replicate 500 mL trays filled with distilled water per cohort and set at a density of 150 larvae per tray) [1]. Larvae were fed a controlled diet of guinea pig chow (PMI Nutrition International, Brentwood, MO USA) and freeze-dried brine shrimp (San Francisco Bay Brand, Newark, CA USA) (4:1). Pupae from each cohort were rinsed in distilled water prior to collecting emerging adults for circulation in the laboratory colony, where adults are maintained on 5% sucrose and rehydrated pesticide-free raisins (Sun-Maid, Fresno, CA USA) provided *ad libitum* [1].

*Iso-female assays*

All handling of the experimental setup was conducted in a biological safety cabinet. Experimental setups for iso-female assays consisted of a nested cage with pupal emergence cups holding 8 mL sterile distilled water and a total of 4 pupae (3x males plus 1x female). Half a sheet of autoclaved Whatman filter paper (Cytiva) was moistened with sterile distilled water and provided for egg-laying (“egg sheet”). Intermediate containers had autoclaved porous stretched fabric suspending a sterile cotton wick saturated with 0.2 μm filter-sterilized 5% sucrose solution. Intermediate containers were placed inside a final chamber for aseptic containment.

*Field-based glycerol stocks*

The field-derived stock used in “*Community passaging under varying larval density*” represented late successional pitcher fluid from unmanipulated pitchers first described in previous work on *W. smithii* top-down effects on *Sarracenia purpurea* microbial communities in the field [2]. Remaining pitcher fluid from unmanipulated pitchers was concentrated using step-wise centrifugation and culturable bacterial density in each stock was determined by plating on R2A prior to use in larval development assays. The target density (~10,000 CFU/mL) was optimized based on prior trials in the laboratory, where higher concentrations required excess inoculation volumes or resulted in high larval mortality.

“Uncolonized Field” and “Colonized Field” glycerol stocks used in “*Mixing of parentally- and environmentally-derived microbial communities*” were generated from the fluid of 22 and 6 recently-opened pitcher plants collected in Cedarburg Bog in Saukville, WI USA (43°23.2′ N, 88°0.63′ W) [3, 4]. Young pitchers were determined to be recently-opened by inspecting color and plant tissue rigidity [5–7]. Pitcher fluid was then harvested and transported on ice back to the laboratory, where fluid was visually inspected for the presence of macroinvertebrate larvae of any kind. Pitchers determined to contain background macroinvertebrate colonization were used to generate “Colonized” stocks and the remainder used to generate “Uncolonized” stocks. Field-derived stocks were prepared through step-wise centrifugation and culturable bacterial density in each stock was determined by plating on R2A prior to use in larval development assays. Wells inoculated with field-derived stocks were also normalized to ~900,000 CFU/mL (average culturable bacterial density of uncolonized young pitchers in the field) (**Supp Fig. 5**) [5–7]. Due to differing stock concentrations, varying volumes of glycerol inoculant were required to establish starting communities of equal bacterial density (16-41 μL), resulting in marginal differences in carry-over material from stock sources that did not correspond with developmental outcomes in recipient larvae.

*DNA extraction, library preparation, and sequence processing*

Whole adult mosquito samples generated from iso-female assays (“mated males” and “mothers”) were frozen at -20 °C at the time of collection. Adults were thawed and wings mounted and measured immediately prior to processing for DNA extraction as follows. Adults were surface sterilized by submerging adults in EtOH (70%) and bleach solution (0.05%) prior to six washes in sterile water (Corning, Corning, NY USA). Pools of mated males (x3) and single mated females were then homogenized with sterile pestles in lysis buffer (Qiagen) and incubated in proteinase K to enhance total DNA yield from low template samples [8]. Larval samples (larval passaging experiments) were frozen at -20 °C at the time of collection and rinsed six times in sterile water (Corning, Corning, NY USA) prior to homogenization with sterile pestles in lysis buffer. Pellets for all water samples collected were generated by centrifugation to concentrate large volumes to 1 mL (20 min; 3000 rcf) followed by pelleting at high speed (20 min; 20,000 rcf). Supernatants were then decanted prior to storage at -20 °C. Total genomic DNA was then extracted from preserved pellets and homogenates using standard phenol-chloroform methods [7, 8] prior to one-step PCR amplification of the V4 region of the bacterial 16S rRNA gene using barcoded primers (515F/806R) and paired-end sequencing (2 × 250-bp) on an Illumina MiSeq as previously described [1, 10]. Read-joining and filtering were conducted for sequence data derived from iso-female assays and larval passaging assays, as previously described [1], prior to sequence denoising and trimming of paired-end joined reads using Deblur [11]. Processing of sequencing data derived from mixed community inocula assays was conducted analogously with the exception of read-joining due to low reverse read quality. For all data, taxonomy was assigned using a Naïve-Bayes classifier trained against the Greengenes2 database (2022.10) [11, 12].

Subsequent multiple sequence alignment and phylogenetic tree construction were conducted using MAFFT and FastTree2 [13, 14]. All analyses downstream were carried-out using the “phyloseq” package in R (version 4.1.1) [15]. The initial phyloseq objects were decontaminated using frequency- and prevalence-based metrics relative to sequencing and extraction controls implemented in the R package “decontam” [16]. Contaminant reads, reads classified as “chloroplast” or “mitochondria”, samples with <100 reads, and samples failing to saturate in rarefaction curves were removed prior to conducting downstream analyses.

**Supplemental references**

1. Arellano AA, Coon KL. Bacterial communities in carnivorous pitcher plants colonize and persist in inquiline mosquitoes. *Anim Microbiome* 2022; **4**: 13.

2. Arellano AA, Young EB, Coon KL. An inquiline mosquito modulates microbial diversity and function in an aquatic microecosystem. *Mol Ecol* 2024:e17314.

3. Bott T, Meyer GA, Young EB. Nutrient limitation and morphological plasticity of the carnivorous pitcher plant *Sarracenia purpurea* in contrasting wetland environments. *New Phytol* 2008; **180**: 631–641.

4. Grothjan JJ, Young EB. Diverse microbial communities hosted by the model carnivorous pitcher plant *Sarracenia purpurea* : analysis of both bacterial and eukaryotic composition across distinct host plant populations. *PeerJ* 2019; **7**: e6392.

5. Fish D, Hall DW. Succession and stratification of aquatic insects inhabiting the leaves of the insectivorous pitcher plant, *Sarracenia purpurea*. *Amer Midl Naturalist* 1978; **99**: 172.

6. Istock CA, Vavra KJ, Zimmer H. Ecology and evolution of the pitcher-plant mosquito. 3. resource tracking by a natural population. *Evolution* 1976; **30**: 548.

7. O’Meara GF, Lounibos LP, Brust RA. Repeated Egg Clutches without Blood in the Pitcher-Plant Mosquito13. *Ann Entomol* 1981; **74**: 68–72.

8. Brettell LE et al. Mosquitoes reared in nearby insectaries at the same institution have significantly divergent microbiomes. *Environmental Microbiology* 2025;**27**.

9. Sambrook J, Fritsch EF, Maniatis T. *Molecular cloning: a laboratory model*, 2nd ed. 1989. Cold Spring Harbor Laboratory Press, Cold Spring Harbor, NY.

10. Stevenson DM, Weimer PJ. Dominance of *Prevotella* and low abundance of classical ruminal bacterial species in the bovine rumen revealed by relative quantification real-time PCR. *Appl Microbiol Biotechnol* 2007; **75**: 165–174.

11. Kozich JJ, Westcott SL, Baxter NT, Highlander SK, Schloss PD. development of a dual-index sequencing strategy and curation pipeline for analyzing amplicon sequence data on the MiSeq Illumina sequencing platform. *Appl Environ Microbiol* 2013; **79**: 5112–5120.

12. Amir A, McDonald D, Navas-Molina JA, Kopylova E, Morton JT, Zech Xu Z, et al. Deblur rapidly resolves single-nucleotide community sequence patterns. *mSystems* 2017; **2**.

13. McDonald D, Jiang Y, Balaban M, Cantrell K, Zhu Q, Gonzalez A, et al. Greengenes2 enables a shared data universe for microbiome studies. 2022. *Bioinformatics*.

14. Pedregosa F, Varoquaux G, Gramfort A, Michel V, Thirion B, Grisel O, et al. Scikit-learn: machine learning in python. *J Mach Learn* 2012; **12**: 2825–2830.

15. Katoh K, Standley DM. MAFFT multiple sequence alignment software version 7: improvements in performance and usability. *Mol Biol Evol* 2013; **30**: 772–780.

16. Price MN, Dehal PS, Arkin AP. FastTree 2 – Approximately maximum-likelihood trees for large alignments. *PLoS ONE* 2010; **5**: e9490.

17. McMurdie PJ, Holmes S. phyloseq: An R package for reproducible interactive analysis and graphics of microbiome census data. *PLoS ONE* 2013; **8**: e61217.

18. Davis NM, Proctor DM, Holmes SP, Relman DA, Callahan BJ. Simple statistical identification and removal of contaminant sequences in marker-gene and metagenomics data. *Microbiome* 2018; **6**: 226.

**Supplemental figure captions and table names**

**Supp Fig 1.** Iso-female assay summary data for adult mosquito mothers depicting variation in (A) wing size, (B) egg hatch water culturable bacterial density, (C) number of eggs laid, and (D) proportion of eggs that hatched. Boxplots show high, low, and median values, with lower and upper edges of each box denoting first and third quartiles, respectively. Bacterial density was determined by dilution plating on solid R2A media and counting colony forming units (CFUs). Colony counts are log10 transformed for visualization. Bivariate effect plots of mixed-effects logistic regressions correlating hatch probability (E) and survival probability (F) with egg water bacterial density. Regressions also include maternal wing length as a fixed effect and trial as a random effect. Shaded regions indicate 95% confidence intervals with fixed effect coefficient estimates and *P* values displayed

**Supp Fig 2.** (A) Distribution of time to pupation (days) for all iso-female development assays. (B) Distribution of female (*left*) and male (*right*) offspring wing lengths (mm) for all iso-female development assays. Neither time to pupation nor wing lengths differed between independent trials, which are represented as pooled in both panels. Boxplots show high, low, and median values, with lower and upper edges of each box denoting first and third quartiles, respectively.

**Supp Fig 3.** (A) Beta diversity of larval passaging samples (bacterial pellet from late larval water or 2-10 homogenized larvae) relative to the inoculating community stock faceted by timepoint (48 hr and 12 day). Beta diversity was determined as the Euclidean distance between samples using a center log ratio (clr)-transformed feature table (Aitchison distance). (B) Culturable bacterial density from late larval water. Bacterial density was determined by dilution plating on solid R2A media and counting colony forming units (CFUs). Colony counts are log10 transformed for visualization and to meet the assumptions of parametric statistics. Boxplots show high, low, and median values, with lower and upper edges of each box denoting first and third quartiles, respectively. Different letters indicate significant differences between treatment groups (Tukey–Kramer HSD test, *P*<0.05).

**Supp Fig 4.** Relative abundance of bacterial genera present in community inoculation stocks derived from the laboratory setting. Bars of a given color represent the proportion of sequencing reads assigned to a given genus in a glycerol-preserved homogenate sample. The most common genera are represented for each of three focal phyla with a base color. Less common genera are collapsed to the category “Other”.

**Supp Fig 5.** (A) Relative abundance of bacterial genera present in community inoculation assays faceted by treatment. Bars of a given color represent the proportion of sequencing reads assigned to a given genus in a late larval water sample for a given well of a six-well plate gnotobiotic development assay. The most common four genera are represented for each of four focal phyla with a base color. Less common genera are collapsed to the category “Other”. (B) Proportion of larvae in each gnotobiotic community background which survived the 27 day period in which development was recorded. (C) Proportion of larvae in each gnotobiotic community background which pupated the 27 day period in which development was recorded. (D) Ridgeline stacked density plots for time to pupation across gnotobiotic community backgrounds. Curve height indicates a greater number of individuals pupating at a given point along the horizontal axis. Inoculation source (Parental, Uncolonized, mixed inocula of Parental and Uncolonized, or Colonized) is indicated by color. Asterisks indicate significant differences in time to pupation (Dunn’s test). * *P*<0.05, ** *P*<0.01, *** *P*<0.001

**Supp Fig 6.** Effect plot depicting differentially abundant bacterial ASVs enriched in pairwise comparison of late larval water from gnotobiotic assay wells inoculated with field-derived communities with or without prior mosquito visitation. Each point represents a bacterial ASV and colored points were conservatively identified as differentially abundant. Points are colored by phylum. Dashed lines represent equal difference (between group variation) and dispersion (within group variation). 

**Supp Fig 7.** Early pitcher fluid culturable bacterial density. Boxplots show high, low, and median values, with lower and upper edges of each box denoting first and third quartiles, respectively. Bacterial density was determined by dilution plating on solid R2A media and counting colony forming units (CFUs). Colony counts are log10 transformed for visualization and to meet the assumptions of parametric statistics. Non-significant Welch’s *t*-test result reported comparing pitchers with documented prior visitation by *W. smithii* (Colonized) and those without (Uncolonized).

**Supplemental Table 1.** Sample detail and alpha diversity for iso-female assay samples

**Supplemental Table 2.** Taxonomy and logistic regression model coefficients for significant bacterial correlates of development in iso-female assays

**Supplemental Table 3.** Sample detail and alpha diversity for larval passaging assay samples

**Supplemental Table 4.** Summary of alpha diversity comparisons among water and larval samples from larval passaging assays

**Supplemental Table 5.** Beta diversity summary of variation in composition (PERMANOVA) and heterogeneity (PERMDISP) of larval passaging samples

**Supplemental Table 6.** Taxonomy, differential abundance, and logistic regression coefficient of larval survival for larval passaging bacteria

**Supplemental Table 7.** Sample detail and alpha diversity for gnotobiotic assay samples

**Supplemental Table 8.** Summary of alpha diversity comparisons among water samples from gnotobiotic assays

**Supplemental Table 9.** Beta diversity summary of variation in composition (PERMANOVA) and heterogeneity (PERMDISP) of gnotobiotic assay samples

**Supplemental Table 10.** Taxonomy and differential abundance of late larval water bacteria by gnotobiotic inoculum condition
